# A novel diagnostic algorithm equipped on an automated hematology analyzer to differentiate between common causes of febrile illness in Southeast Asia

**DOI:** 10.1371/journal.pntd.0007183

**Published:** 2019-03-14

**Authors:** Susantina Prodjosoewojo, Silvita F. Riswari, Hofiya Djauhari, Herman Kosasih, L. Joost van Pelt, Bachti Alisjahbana, Andre J. van der Ven, Quirijn de Mast

**Affiliations:** 1 Health Research Unit, Faculty of Medicine, Universitas Padjadjaran, Bandung, Indonesia; 2 Department of Internal Medicine, Hasan Sadikin General Hospital, Bandung, Indonesia; 3 Indonesia Research Partnership of Infectious Disease (INA-RESPOND), Jakarta, Indonesia; 4 Department of Laboratory Medicine, University Medical Centre Groningen, Groningen, The Netherlands; 5 Department of Internal Medicine, Radboud Center for Infectious Diseases, Radboud university medical center, Nijmegen, The Netherlands; Mahidol Univ, Fac Trop Med, THAILAND

## Abstract

**Background:**

Distinguishing arboviral infections from bacterial causes of febrile illness is of great importance for clinical management. The Infection Manager System (IMS) is a novel diagnostic algorithm equipped on a Sysmex hematology analyzer that evaluates the host response using novel techniques that quantify cellular activation and cell membrane composition. The aim of this study was to train and validate the IMS to differentiate between arboviral and common bacterial infections in Southeast Asia and compare its performance against C-reactive protein (CRP) and procalcitonin (PCT).

**Methodology/Principal findings:**

600 adult Indonesian patients with acute febrile illness were enrolled in a prospective cohort study and analyzed using a structured diagnostic protocol. The IMS was first trained on the first 200 patients and subsequently validated using the complete cohort. A definite infectious etiology could be determined in 190 of 463 evaluable patients (41%), including 89 arboviral infections (81 dengue and 8 chikungunya), 94 bacterial infections (26 murine typhus, 16 salmonellosis, 6 leptospirosis and 46 cosmopolitan bacterial infections), 3 concomitant arboviral-bacterial infections, and 4 malaria infections. The IMS detected inflammation in all but two participants. The sensitivity, specificity, positive predictive value (PPV), and negative predictive value (NPV) of the IMS for arboviral infections were 69.7%, 97.9%, 96.9%, and 77.3%, respectively, and for bacterial infections 77.7%, 93.3%, 92.4%, and 79.8%. Inflammation remained unclassified in 19.1% and 22.5% of patients with a proven bacterial or arboviral infection. When cases of unclassified inflammation were grouped in the bacterial etiology group, the NPV for bacterial infection was 95.5%. IMS performed comparable to CRP and outperformed PCT in this cohort.

**Conclusions/Significance:**

The IMS is an automated, easy to use, novel diagnostic tool that allows rapid differentiation between common causes of febrile illness in Southeast Asia.

## Author summary

Distinguishing arboviral infections, such as dengue, from bacterial causes of febrile illness is of great importance for clinical management and antimicrobial stewardship. In resource-limited countries, costly and expertise-reliant diagnostic assays cannot be performed routinely. The Infection Manager Software (IMS) is a novel diagnostic algorithm equipped on an automated Sysmex hematology analyzer, making use of the principle that different infections evoke different changes in blood cell number and cell phenotype. In a cohort of adult Indonesian patients presenting to hospital with an arboviral and/or bacterial infection, we first trained and subsequently evaluated the diagnostic performance of the IMS to distinguish common causes of acute febrile illness. The authors show that the IMS has a reasonable sensitivity for detection of arboviral and bacterial infections and a high specificity. In comparison with the commonly used biomarkers C-reactive protein (CRP) and procalcitonin (PCT), the performance of the IMS was comparable to CRP and better than PCT. The authors conclude that the IMS is a novel, automated, easy to use diagnostic tool that allows rapid differentiation between common causes of febrile illness in Southeast Asia.

## Introduction

Arboviruses and bacterial infections such as salmonellosis, leptospirosis, and rickettsiosis are common causes of acute febrile illness in tropical and subtropical countries [1–3]. Discriminating between these infections is of great importance to triage patients in need of antibiotics or monitoring for dengue complications. In daily practice, dengue and bacterial infections are often diagnosed on clinical grounds and many patients are prescribed antibiotics without laboratory confirmation of a bacterial infection. Confirmatory microbiological tests, including blood cultures, serology, molecular tests, and antigen- or antibody-based rapid tests are frequently unavailable and suffer from important diagnostic limitations.

An alternative for pathogen-specific diagnostic tests is the assessment of the host immune response, using biomarkers such as C-reactive protein (CRP) or procalcitonin (PCT) [4, 5]. Disease-specific changes in circulating blood cells may also be helpful, for example, leukopenia and thrombocytopenia support a diagnosis of dengue [6]. The discriminatory performance of cell numbers alone is, however, insufficient for clinical decision-making. A promising development is the ability to measure phenotypic changes in blood cells by automated hematology analyzers. For example, activated leukocytes contain more lipid rafts in their cell membrane and altered intracellular DNA/RNA levels [7] which can be quantified using specific reagents and distinct fluorescence patterns [8, 9].

Based on the principle that different infections evoke different patterns in blood cell number and phenotype, a diagnostic algorithm called the Infection Manager System (IMS), was developed for use on Sysmex hematology analyzers. The IMS indicates whether an inflammatory response is present and whether an arboviral, bacterial, or malarial origin is suspected. The aim of our present study was to enroll adult patients with common causes of undifferentiated fever in Southeast Asia in order to train and evaluate the diagnostic performance of the IMS for these infections, as well as to compare the diagnostic performance against CRP and PCT.

## Methods

### Design and study population

A prospective cohort study was conducted between July 2014 and February 2016 in three hospitals (Hasan Sadikin University Hospital, Salamun General Hospital, and Cibabat General Hospital) and two primary care outpatient clinics, all located in Greater Bandung, the capital of the West Java province in Indonesia. Patients aged 14 years and above presenting an acute febrile illness and clinical suspicion of an arboviral infection, salmonellosis, leptospirosis, rickettsiosis, or any other common bacterial infection were enrolled. Exclusion criteria included pregnancy and the suspicion of a chronic infection, such as tuberculosis or HIV, and severe concomitant conditions like dialysis, autoimmune diseases, or malignancies. The sample size of 600 individuals was based on the assumption that a proven or probable bacterial or arboviral infection could be diagnosed in 50% of enrolled patients and that enteric fever, leptospirosis, or rickettsiosis could be diagnosed in approximately 20% (n = 30) of subjects with a proven or probable bacterial infection.

To test how often the IMS flags an inflammatory response in healthy adults, the trained IMS was also tested in a cohort of healthy Dutch adults, derived from a well-established prospective population-based study, incorporating 13,432 individuals from the north of the Netherlands (www.lifelines.nl).

### Study procedures

The first selection of patients was done by treating physicians at the participating health facilities on the basis of clinical features and routine additional examinations. Demographic data, medical history, physical examination, results of laboratory and radiology tests, and suspected diagnosis were recorded in a standardized electronic study case report form. All admitted patients were followed up three days after enrolment to evaluate the clinical picture and perform additional diagnostic tests on indication. A policlinic visit was planned with the same purpose between days 7–14 after enrolment day. Non-admitted patients were followed up twice: first appointment between 2–7 days after enrolment, a second appointment within one week thereafter.

### Diagnostic procedures and case definitions

**Fig 1:**
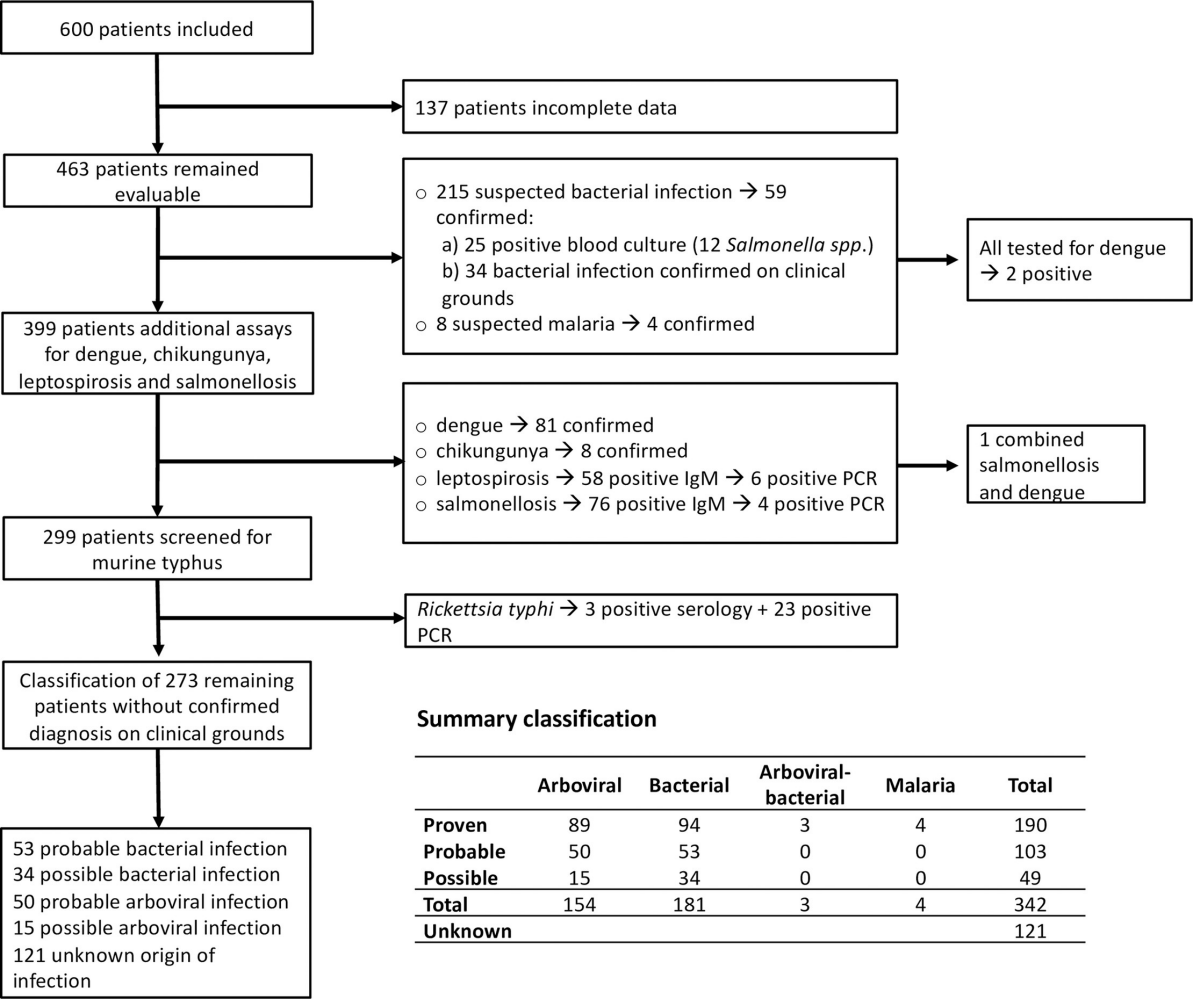
Patient flow chart and classification of patients.

Fig 1 summarizes the study flow and diagnostic procedures. Blood was drawn at inclusion in all patients for immediate hemocytometry and microbiological testing. EDTA plasma, serum, and whole blood were stored at -80°C for additional microbiological tests. Initial microbiological tests were performed at the discretion of the treating physician. These included the performance of blood cultures in patients with a suspected bacterial sepsis or enteric fever, pus cultures in case of an abscess, and dengue NS1 rapid test or serological tests for suspected dengue, enteric fever, or leptospirosis. Radiological examinations such as a chest X-ray were performed on indication.

Next, stored blood of all enrolled subjects was tested using the following diagnostic algorithm: dengue diagnostics were performed using a dengue NS1 antigen rapid diagnostic test (RDT), and if negative, paired dengue IgM and IgG serology and dengue PCR. Furthermore, RDTs or serology were done on all samples for chikungunya IgM, Salmonella IgM (Tubex^®^), and Leptospira IgM (Panbio^®^). In case of a positive chikungunya IgM, Salmonella IgM score ≥4 or a positive Leptospira IgM, specific serum or whole blood PCRs for these pathogens were performed. The remaining cases without a proven diagnosis were tested for *Rickettsia typhi* IgM and IgG, followed by a specific *R*. *typhi* real-time PCR in case of a positive result.

The following case definitions were used: a proven dengue virus infection was defined as: i) positive result of NS1 RDT or dengue PCR, or ii) seroconversion of anti-dengue IgM and/or IgG, or iii) fourfold or greater increase of anti-dengue IgG titers in convalescent serum. Chikungunya or Leptospirosis were proven when the PCR was positive. Salmonellosis was proven when *Salmonella* spp. were isolated from blood culture or when the whole blood Salmonella PCR was positive. Murine typhus was proven when there was seroconversion or a four-fold increase in IgM or IgG *R*. *typhi* titer or a positive PCR on the buffy coat. A proven cosmopolitan bacterial infection was defined as isolation of a pathogenic pathogen from blood culture or other sterile location, or by a combination of clinical features and results of radiology, for example in case of pneumonia. Malaria was proven if *Plasmodium* parasites were detected on a blood smear.

In case no proven diagnosis was obtained, two experienced clinicians (AvdV and QdM) graded the remainder of the cases as probable or possible arboviral or bacterial infection without any further sub-classification or as fever from unknown origin. Grading was done using all clinical data and additional investigations, but without results of IMS and CRP or PCT.

### Laboratory procedures

Hemocytometry was done on EDTA blood within 4 hours using Sysmex XN-1000, Sysmex XN-550, and a regular Sysmex XE-5000 analyzer. Details of the performed microbiological tests and the CRP and PCT measurements are given in S1 Table.

### Infection Manager System and analysis

The IMS is based on novel techniques that quantify cellular activation and cell membrane composition using distinct fluorescence and surfactant reagents that target RNA, DNA, and bioactive lipids, respectively [8–10]. The IMS algorithm is given in S1 Fig. The IMS first flags whether an inflammatory response is detected and if so, whether it fits a bacterial, (arbo)viral, or malarial origin or cannot be classified and designated as an unspecified inflammatory response. When no inflammatory reaction is noticed, no message is given.

### Analysis and role of the sponsor

The sponsor was not involved in data acquisition, including results of hemocytometry or microbiological assays. Employees of the sponsor were involved in the training of the IMS algorithm using the first 200 enrolled cases with the goal to further optimize the IMS performance. For this training, the sponsor had access to clinical information, results from microbiology and radiology examinations, and the tentative cause of the febrile illness as classified by the clinical study team. Results of PCRs and CRP/PCT were not yet available at that time. Next, the final version of the IMS was tested on all evaluable cases with employees of the sponsor classifying all enrolled patients into: no sign of inflammation, or suspected arboviral, bacterial, malarial, or unspecified inflammation. For this classification, the sponsor was blinded to all clinical data, results from additional tests and the final classification by the study team of the cause of the febrile illness. Whereas the IMS classification was performed by the sponsor in this feasibility study, the intention is to create an analyzer that directly reports the IMS classification after measurement of the blood sample without requiring data to be sent to another site for analysis.

For CRP and PCT the following cut-off levels were evaluated in predicting a bacterial etiology of fever: for CRP >20 mg/L and >40 mg/L and for PCT >0.5 ng/mL and >2.0 ng/mL plasma levels upon admission, respectively [2]. For additional analyses, a special group named ‘antibiotics’, was created, containing individuals who were flagged as either bacterial or unspecified inflammation by the IMS, as antibiotics may be indicated in these cases. Patients with a proven concomitant arboviral-bacterial infection were also classified as bacterial infection.

Descriptive statistics were conducted for all variables. Differences in hematology parameters between groups were analyzed using Wilcoxon rank sum test in case of two groups and Kruskal-Wallis test in case of more than two groups. All statistical analyses were performed using R (R Core Team (2016)).

### Ethics statement

All procedures followed were in accordance with the ethical standards of the Helsinki Declaration. All study participants provided written informed consent. In patients aged 14–18 years, a parent or guardian provided informed consent with written assent by the child. The study protocol was approved by the Ethics Committee of Hasan Sadikin General Hospital (LB.02.01/C02/515/I/2015, LB.02.01/C02/2352/II/2016).

## Results

### Study Subjects and Flow of Patients

A total of 600 patients were enrolled. A total number of 137 patients were subsequently excluded because of missing data, mostly because of insufficient follow-up while no proven diagnosis was made. From the remaining 463 subjects, 342 patients could be classified as having a proven, probable, or possible arboviral, bacterial, combined arboviral-bacterial, or malaria infection (Fig 1). A total number of 89 individuals had a proven arboviral infection: 81 cases with dengue, based on a positive result of a dengue NS1 antigen test (n = 68), IgM dengue seroconversion (n = 9), or dengue PCR (n = 4) and eight cases with chikungunya. Three patients with IgM dengue seroconversion also had a bacteremia (two *Salmonella* spp. and one *Staphylococcus aureus*). A total of 94 patients had a proven bacterial infection: murine typhus (n = 26), salmonellosis (n = 16), leptospirosis (n = 6) and cosmopolitan bacterial infections, including bacteremia (n = 13), community-acquired pneumonia (n = 15), skin or soft tissue infection (n = 11), urinary tract infection (n = 5) and single cases of puerperal infection and peritonitis. A total number of 121 patients were classified as unknown origin of infection.

Baseline characteristics of participants with a proven infection are summarized in Table 1; characteristics of participants with proven or probable infections are given in S2 Table. In total, 82% of the enrolled patients were hospitalized and ten patients died during hospitalization, all from the proven bacterial group.

**Table 1:** Baseline characteristics of cases with a proven infectious etiology.

	All patients (n = 190)	Bacterial etiology (n = 94)	Arboviral etiology (n = 89)	Arboviral-bacterial (n = 3)	Malaria (n = 4)
Age (years)	36 (20;53)	46 (26.5;61.5)	28 (19;43)	17 (8.5;33)	25 (19.8;35)
Male, n (%)	76 (40)	32 (34)	41 (46.1)	0 (0)	3 (75)
Admitted, n (%)	156 (82.1)	73 (77.7)	76 (85.4)	3 (100)	4 (100)
Current fever, n (%)	130 (68.4)	50 (53.2)	73 (82)	3 (100)	4 (100)
Duration of fever (days)	4 (3;6)	4 (3;7)	4 (3;5)	8 (4.5;8.5)	12 (8.5;12)
BMI (kg/m^2^)	21.4 (18.7;23.9)	21.1 (18.7;23.6)	21.6 (18.8;23.8)	20 (18.4;22.5)	23.2 (21.6;25)
Mortality, n (%)	10 (5.3)	9 (9.6)	0 (0)	1 (33.3)	0 (0)
**Routine hematology**					
Leukocytes (10^3^/μL)	5.1 (3.4;9.9)	11.4 (6.2;15.6)	3.6 (2.6;4.8)	3.6 (3.5;4.3)	4.7 (4.6;5.6)
Neutrophils (10^3^/μL)	3 (1.5;7.2)	8.1 (4.6;13.6)	1.5 (1.1;2.6)	2.6 (2.5;2.7)	2.2 (2.1;2.8)
Lymphocytes (10^3^/μL)	1.2 (0.7;1.8)	1.2 (0.6;1.6)	1.3 (0.8;1.8)	0.7 (0.6;1.2)	2.1 (1.7;2.6)
Monocytes (10^3^/μL)	0.4 (0.3;0.7)	0.6 (0.3;1)	0.3 (0.2;0.5)	0.4 (0.2;0.4)	0.5 (0.3;0.6)
Eosinophils (10^3^/μL)	0.01 (0;0.05)	0.01 (0;0.04)	0.01 (0;0.04)	0.02 (0.01;0.08)	0. (0.1;0.1)
Platelets (10^9^/L)	106 (575;200)	156 (102;270)	79 (40;118)	89 (59;96)	75 (56;95)
Hemoglobin (g/dL)	13.4 (11.4;15)	11.6 (9.6;13.4)	14.6 (13.4;15.6)	10.9 (10.4;13)	7.2 (6.4;8)
Hematocrit (%)	38.9 (33.2;43.7)	34 (27.8;39.5)	42.5 (38.4;45.6)	36.2 (32.3;40)	21.6 (18.2;25.3)
**Biomarkers**					
CRP (mg/L)	35 (10;117)	110 (52;192)	11 (5;23)	97 (58;146)	142 (65;202)
PCT (ng/mL)	0.9 (0.3;3.9)	2.6 (0.8;7.5)	0.4 (0.2;0.7)	5 (3.8;8.8)	34.2 (20.7;43)

Data are presented as median (25%; 75% percentile) unless indicated otherwise. CRP, C-reactive protein; PCT, procalcitonin

### Hemocytometry parameters

**Fig 2:**
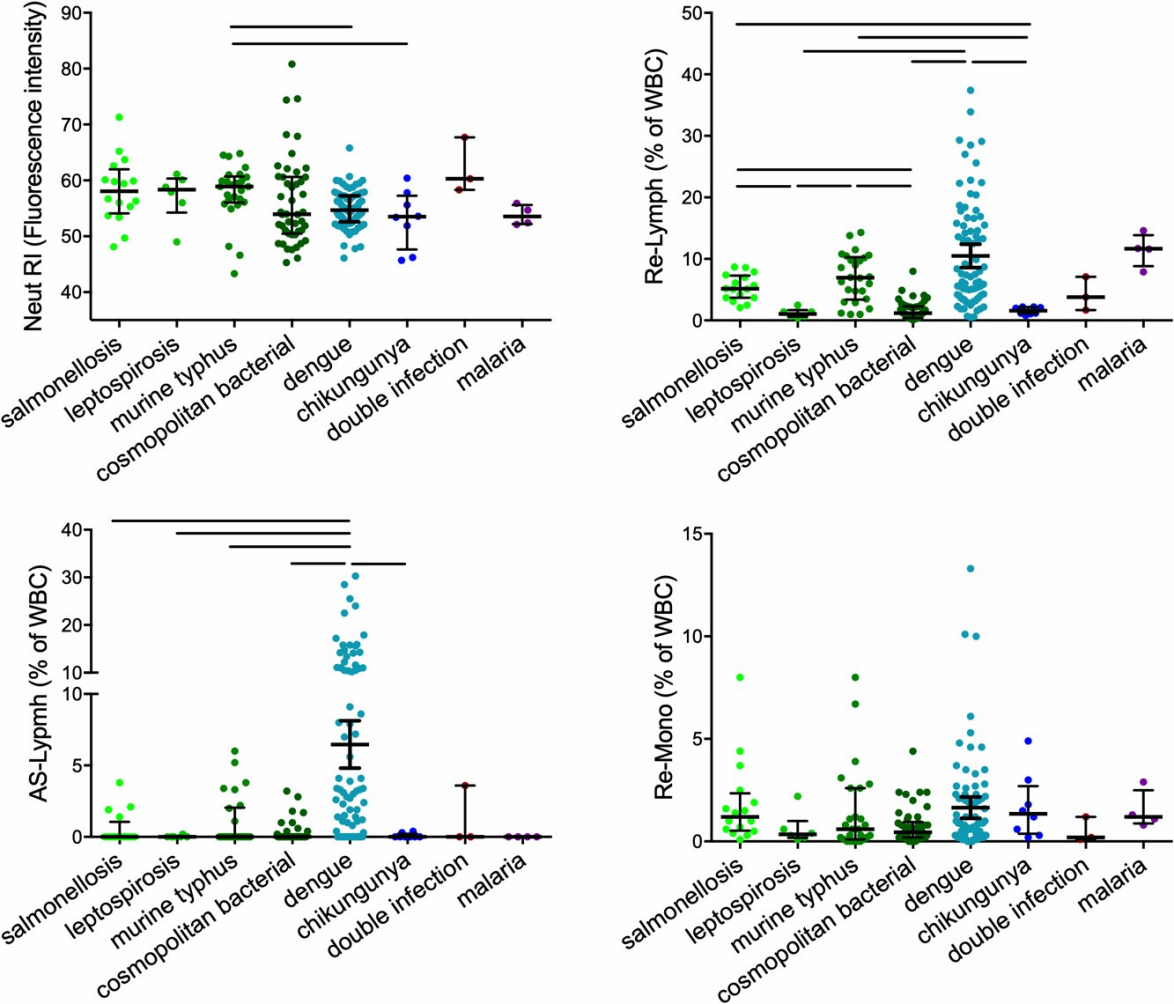
The number or percentage of activated neutrophils (Neut-RI), lymphocytes (Re-Lymph and AS-Lymph), and monocytes (Re-Mono) in patients with a proven infection. The lines indicate median with interquartile ranges. Differences were analyzed using Kruskal Wallis test with post-hoc tests. The lines indicate a statistically significant difference (*P*<0.05) considering correction of the *P* value for multiple testing (Benjamini-Hochberg). WBC, white blood cells.

**Fig 3:**
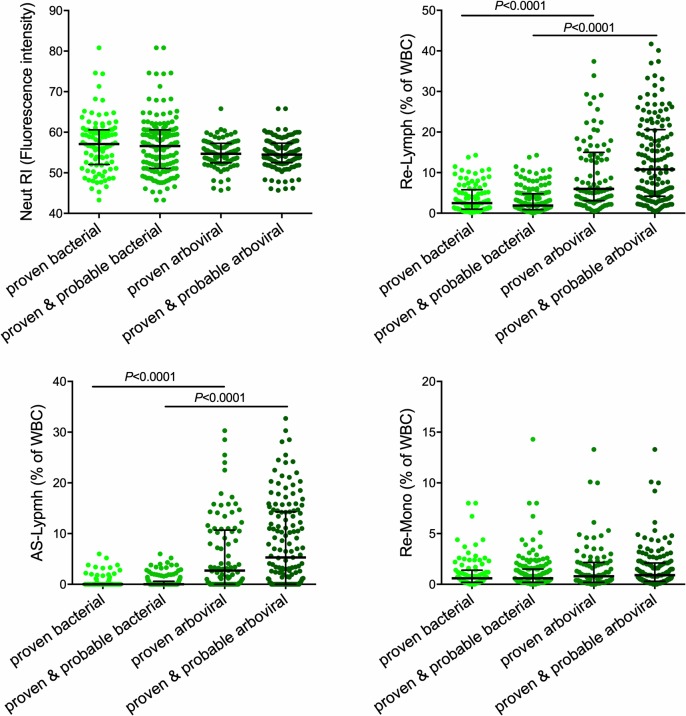
The absolute number or percentage of activated neutrophils (Neut-RI), lymphocytes (Re-Lymph and AS-Lymph) and monocytes (Re-Mono) in patients with proven or proven/probable infections, aggregated in bacterial or arboviral infections. The lines indicate median with interquartile ranges. Differences were analyzed using Kruskal Wallis test with post-hoc tests. WBC, white blood cells.

Fig 2 and Fig 3 show the results of a selection of novel leukocyte parameters per infection or aggregated in arboviral or bacterial infections. Whereas there was a large overlap in the number of activated neutrophils (Neut-RI) and monocytes (Re-Mono) across the different infections, dengue was characterized by a marked increase in AS-Lymph and Re-Lymph, which are considered to represent plasma cells and reactive lymphocytes, respectively. In contrast, chikungunya was not associated with increased AS-Lymph or Re-Lymph. Participants with the intracellular bacterial infections salmonellosis and murine typhus also had significantly higher Re-Lymph than those with other bacterial infections (salmonellosis vs. leptospirosis *P* = 0.006; salmonellosis vs. cosmopolitan bacterial infection *P*< 0.0001; murine typhus vs. leptospirosis *P* = 0.007; murine typhus vs. cosmopolitan bacterial infections *P*< 0.0001).

### Diagnostic performance of IMS

Table 2 summarizes the diagnostic performance of the IMS. An inflammatory response was flagged in all but two cases; one case of dengue in whom the dengue diagnosis was based on IgM seroconversion, and one patient with salmonellosis. Overall, the sensitivity, specificity, positive predictive value (PPV), and negative predictive value (NPV) of the IMS for arboviral infections were 69.7%, 97.9%, 96.9% and 77.3%, respectively, and for bacterial infections 77.7%, 93.3%, 92.4% and 79.8%. Inflammation remained unclassified in 19.1% and 22.5% of patients with a proven bacterial or arboviral infection, respectively. Importantly, six out of seven (86%) cases with proven chikungunya were classified as unspecified inflammation. Similarly, a relatively high proportion of cases with murine typhus were either classified as unspecified inflammation (27%) or arboviral inflammation (8%). None of the other proven or probable bacterial infections were classified as arboviral. The three cases with a combined arboviral-bacterial infection were all flagged as bacterial infection. One of four malaria cases was not correctly flagged as being malaria.

**Table 2:** IMS Classification in proven cases and in combined proven/probable cases.

	Type of inflammation indicated by IMS				
	Arboviral etiology	Bacterial etiology	Unspecified inflammation	Malaria	No inflammation
**Proven infections**					
**Arboviral**	62	6	20	0	1
Dengue	61	6	13	0	1
Chikungunya	1	0	7	0	0
**Bacterial**	2	73	18	0	1
Salmonellosis	0	12	3	0	1
Leptospirosis	0	6	0	0	0
Murine typhus	2	17	7	0	0
Cosmopolitan	0	38	8	0	0
**Arboviral-bacterial**	0	3	0	0	0
**Malaria**	1	0	0	3	0
**Proven/Probable infections**					
Arboviral	105	9	24	0	1
Bacterial	2	116	27	0	2
Arboviral-bacterial	0	3	0	0	0
Malaria	1	0	0	3	0

Data are number.

### Diagnostic performance IMS in comparison to CRP and PCT

**Fig 4:**
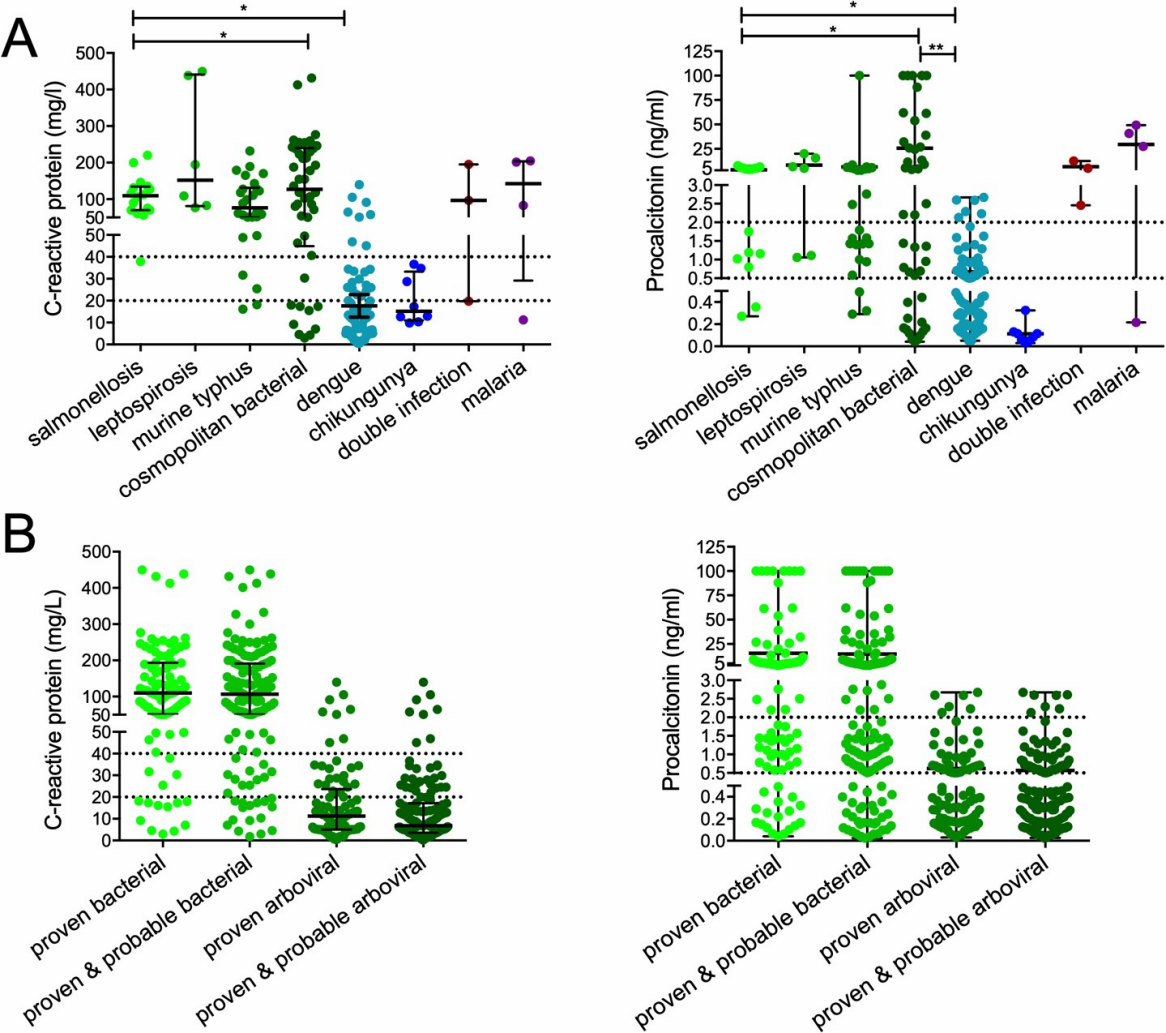
C-reactive protein (CRP) and procalcitonin (PCT) concentrations. (A) enrolled patients with a proven infection aggregated per infection; (B) enrolled patients with a proven or probable infection aggregated in bacterial or arboviral infections. The lines with error bars indicate median with interquartile range. Differences were analyzed using Kruskal Wallis test with post-hoc tests with multiple testing correction (Benjamini-Hochberg). * indicates *P*<0.05.

Fig 4A shows CRP and PCT plasma levels at study enrolment per infection, and Fig 4B provides these levels for cases aggregated in proven or proven/probable bacterial or arboviral etiology. In the proven cases, a bacterial etiology was associated with significantly higher CRP and PCT levels than a proven arboviral etiology with median (IQR) CRP levels of 110mg/L (52-192mg/L) vs. 11mg/L (5-23mg/L; *P*<0.0001) and PCT levels of 2.6ng/mL (0.8–7.5ng/mL) and 0.4ng/mL (0.2–0.7ng/mL; *P*<0.0001), respectively (Table 1 and Fig 4A). Table 3 summarizes the diagnostic performance of the IMS compared with CRP and PCT. A special category, named ‘antibiotics’, was created for the IMS result, containing individuals who were flagged as either bacterial or unspecified inflammation by the IMS, as antibiotics may be indicated in these. In total, 88% and 84% of bacterial cases had CRP levels above the pre-defined cut-offs of >20mg/L or >40mg/L, respectively, whereas 81% and 54% had PCT levels >0.5ng/mL or >2.0ng/mL, respectively. For the arboviral group, 72% and 91% of cases had CRP levels below these cut-offs and 55% and 93% PCT levels below these cut-offs, respectively. The optimal CRP plasma level cut-off to distinguish between a bacterial and viral etiology was 36.6 mg/L (sensitivity 85.1% with specificity 91.0%; area under the receiver operating characteristic (ROC) curve 0.92) and for PCT 0.96ng/mL (sensitivity 72.3%; specificity 83.1%; area under the ROC curve 0.81). Overall, CRP with a cut-off of 40mg/L had a somewhat higher sensitivity for bacterial infections than the IMS with a somewhat lower specificity. Using the ‘antibiotics’ classification in IMS shifted the balance to a higher sensitivity and higher NPV, but lower specificity compared with CRP. PCT performed less well than either the IMS or CRP.

**Table 3:** Diagnostic performance of the IMS compared with CRP and PCT.

	Bacterial etiology, n (%)	Arboviral etiology, n (%)	Sensitivity	Specificity	PPV	NPV
**Proven infections**						
**IMS**						
bacterial	73/94 (77.7)	6/89 (6.7)	77.7%	93.3%	92.4%	79.8%
arboviral	2/94 (2.1)	62/89 (69.7)	69.7%	97.9%	96.9%	77.3%
unspecified	18/94 (19.1)	20/89 (22.5)				
no inflammation	1/94 (1.1)	1/89 (1.1)				
‘antibiotics’*	91/94 (96.8)	26/89 (29.2)	96.8%	70.8%	77.8%	95.5%
**CRP > 20mg/L**	83/94 (88.3)	25/89 (28.1)	88.3%	71.9%	76.9%	85.3%
**CRP > 40mg/L**	79/94 (84.0)	8/89 (9.0)	84.0%	91.0%	90.8%	84.4%
**PCT > 0.5ng/mL**	76/94 (80.9)	40/89 (44.9)	80.9%	55.1%	65.5%	73.1%
**PCT > 2.0ng/mL**	51/94 (54.3)	6/89 (6.7)	54.3%	93.3%	89.5%	65.9%
**Proven/probable infections**						
**IMS**						
bacterial	116/147 (78.9)	9/139 (6.5)	78.9%	93.5%	92.8%	80.7%
arboviral	2/147 (1.4)	105/139 (75.5)	75.5%	98.6%	98.1%	81.0%
unspecified	27/147 (18.4)	24/139 (17.3)				
no inflammation	2/147 (1.4)	1/139 (0.7)				
‘antibiotics’*	143/147 (97.3)	33/139 (23.7)	97.3%	76.3%	81.3%	96.4%
**CRP > 20mg/L**	131/147 (89.1)	31/139 (22.3)	89.1%	77.7%	80.9%	87.1%
**CRP > 40mg/L**	122/147 (83.0)	8/139 (5.8)	83.0%	94.2%	93.8%	84.0%
**PCT > 0.5ng/mL**	114/147 (77.6)	56/139 (40.3)	77.6%	59.7%	67.1%	71.6%
**PCT > 2.0ng/mL**	71/147 (48.3)	7/139 (5.0)	48.3%	95.0%	91.0%	63.5%

* The category ‘antibiotics’ are the cases in which the IMS indicates a bacterial infection or unspecified inflammation, as antibiotics may be considered in these cases.

Malaria and double infections were excluded in this analysis due to the small sample size.

CRP, C-reactive protein; PCT, procalcitonin; PPV, positive predictive value; NPV, negative predictive value

### Healthy population cohort

Finally, we determined how frequently the IMS flags an inflammatory response in healthy individuals. A total of 13,432 Dutch subjects were available from the lifelines cohort that had no sign or symptoms of illness or abnormality on routine laboratory examination and in whom IMS data were accessible as well. The IMS indicated an unspecified inflammatory response in five participants.

## Discussion

The main finding of the present study is that a novel diagnostic algorithm operating on an automated Sysmex hematology analyzer, called the IMS, is capable of confirming the presence of an infection in Indonesian adults presenting with an acute febrile illness and discriminate arboviral from bacterial infections.

The IMS is based on the principle that pathogens induce specific changes in the number and phenotype of circulating blood cells and that these changes can differentiate viral from bacterial infections. The idea that algorithms incorporating novel blood count parameters may be used as decision tools for antibiotic therapy is supported by recent studies in febrile children [9] and ICU patients [11, 12]. In resource-limited countries, costly and expertise-reliant diagnostic assays cannot be performed routinely. The IMS has the advantage that it operates on a standard hematology analyzer with results being available within a few minutes at an affordable price. In health facilities with a hematology analyzer, the IMS holds promise as an alternative for pathogen-specific RDTs or host biomarker tests, and as a tool for a more targeted use of pathogen-specific diagnostic assays. In addition, in patients with dengue, daily hemocytometry is advised to monitor platelet and leukocyte counts. This offers a unique opportunity to combine diagnostics with clinical monitoring.

The arboviral group in our study mainly comprised of dengue cases. Dengue is the most common arboviral infection with more than one third of the world's population living in areas at risk for infection [13]. Dengue was characterized by increases in antibody synthesizing (AS-Lymph) and reactive lymphocytes (Re-Lymph), in combination with thrombocytopenia and a high immature platelet fraction. Polyclonal plasmacytosis has previously been reported to be a feature of dengue infections [14, 15]. In chikungunya cases, elevations in AS-Lymph and Re-Lymph were not observed and 86% of chikungunya infections were classified as ‘unspecified inflammation’. The diagnostic performance of the IMS for viral infections other than dengue, including common respiratory infections and other arboviruses such as Zika, therefore awaits to be determined.

Bacterial infections were also aggregated into one group because of relatively low numbers per group. Interestingly, *Salmonella* spp. and *R*. *typhi* are intracellular growing bacteria and infections with these pathogens elicited a distinct pattern with a significantly higher Re-Lymph. Therefore, our data suggest that the IMS also has the potential to differentiate among specific subtypes of bacterial infections.

IMS classified a substantial number of infections as ‘unspecified’ inflammation. Because antimicrobial therapy may still be warranted in conditions flagged as unspecified inflammation, a category ‘antibiotics’ was created. The NPV of the IMS for the ‘antibiotics’ category was high (95.5%), suggesting that the IMS holds promise to improve the correct use of antibiotics as well as antimicrobial stewardship in these settings. Dengue-bacterial co-infections are probably underestimated and withholding antibiotics may have severe consequences [16]. Fortunately, in the three patients with a proven double infection in our study, the IMS scored all as bacterial infections. The IMS can also provide an indication on the presence of malaria, but novel techniques using laser technologies and reagents specifically designed for malaria detection using Sysmex analyzers are currently under clinical evaluation (ClinicalTrials.gov Identifier: NCT02669823).

Overall, the trained IMS performed comparable to CRP with the latter having a slightly higher sensitivity but lower specificity to diagnose bacterial infections. Including cases with unclassified inflammation in the bacterial etiology group (‘antibiotics’ category), the balance shifted to a higher sensitivity, but lower specificity. Cut-offs for clinical decision making depend on the clinical setting. So far, only a few studies have reported CRP or PCT levels in tropical infections [2, 17]. Our findings are comparable to those by Wangrangsimakul et al. who also found a CRP level of 36mg/L as the optimal cut-off level to distinguish between bacterial and viral causes of undifferentiated fever in Thailand [2].

We enrolled patients suspected of having specific infections that are very common throughout much of Southeast Asia (e.g. dengue, enteric fever, leptospirosis, murine typhus) and our findings are therefore most likely applicable to areas outside Indonesia. The performance of the IMS in areas with a different infection epidemiology is currently unknown. Results of a diagnostic study investigating the performance of the IMS in Sub-Saharan Africa are expected in the coming year (ClinicalTrials.gov, NCT02669823). The IMS software operates on routine hematology analyzers (Sysmex XN series) and results are provided within one minute. The costs associated with the assay are expected to be in the range of a regular full blood count. A full blood count is among the most commonly performed laboratory tests–also in resource-poor areas in Asia–and introduction of the IMS algorithm is especially promising for the workup of febrile patients in larger healthcare facilities where hemocytometry analyzers are already in routine use, but which lack facilities for more specialized microbiological assays.

Limitations of the present study are that proof of infection, using microbiology or imaging studies, was obtained in only 35% of cases. Our results do not however differ very much from other similar studies in low-income settings [18, 19]. Secondly, we used stringent microbiological criteria. Despite our efforts to include as much ‘tropical’ infections as possible, the total number of proven tropical bacterial infections remained limited. In line with other studies, we also found that murine typhus is an important and often unrecognized infection [2, 20, 21]. Thirdly, our study did not include consecutive febrile patients, but limited selection to those patients suspected of having a specific type of infection in order to train the IMS algorithm. This, together with the stringent microbiological criteria, may have led to selection bias, e.g. dengue patients of whom the majority had a positive NS1 antigen test. Confirmatory validation studies enrolling consecutive febrile patients are therefore required. Lastly, a cohort of healthy Dutch instead of Indonesian individuals was used to test how frequently the trained IMS indicates inflammation in absence of an infection. Inclusion of a large control population from the same demography would have been preferred, because factors such as ethnicity and living conditions may influence hematological reference ranges. Nonetheless, earlier data showed that reference ranges on Sysmex analyzers in a Dutch and Asian (Indian) population of healthy adults were fairly similar [22, 23], suggesting that important differences in IMS performance are not expected. Age-related differences in reference ranges are bigger, especially between children below the age of six years and adults. Our study did not include children and it is important to emphasize that the IMS first needs validation in children as well as other healthy and patient populations in other areas before it can be introduced on a routine basis.

In conclusion, the IMS is a promising novel diagnostic algorithm that can be equipped on a standard hematology analyzer and can be used to triage patients in need of antibiotics or monitoring for dengue complications.

## Supporting information

S1 Table Diagnostic tests used. (DOCX)

S2 Table Baseline characteristics of proven-probable cases combined. (DOCX)

S1 Fig IMS algorithm. (DOCX)

S1 Checklist STROBE checklist. (DOC)
