# Supplementary material for: A novel diagnostic algorithm equipped on an automated hematology analyzer to differentiate between common causes of febrile illness in Southeast Asia
Source: PLoS Negl Trop Dis. 2019 Mar 14;13(3):e0007183. doi: 10.1371/journal.pntd.0007183 (PMC6435198; doi:10.1371/journal.pntd.0007183)
Supplement: S1 Table — (DOCX) [file pntd.0007183.s001.docx]

**Supplemental information**

**S1 table. Diagnostic tests used.**

| **Test** | **Sample** | **Kit; Laboratory** |
| --- | --- | --- |
|  |  |  |
| Blood culture | Whole blood | BacT^®^/ALERT^®^ Culture Media with Vitek 2 system, bioMérieux, France; performed in laboratory of microbiology, Clinical Pathology Department, Hasan Sadikin General Hospital and BLK |
| Dengue NS1 antigen | Serum | Panbio^®^ Dengue Early Rapid, Alere, US; performed in laboratory of serology, Clinical Pathology Department, Hasan Sadikin General Hospital |
| IgM and IgG Dengue | Serum | Panbio^®^ Dengue Duo Cassette IgM IgG, Alere, US; performed in laboratory of serology, Clinical Pathology Department, Hasan Sadikin General Hospital |
| IgM Chikungunya | Serum | In house CDC Protocol; performed in laboratory of Emerging Virus Research Unit, Eijkman Institute for Molecular Biology, Jakarta |
| Arboviral PCR | Serum | Bio-Rad real-time PCR, US; performed in laboratory of Emerging Virus Research Unit, Eijkman Insitute for Molecular Biology, Jakarta |
| IgM Leptospira | Serum | Panbio^®^ Leptospira IgM ELISA, Alere, US; performed in laboratory of serology, Clinical Pathology Department, Hasan Sadikin General Hospital |
| IgM Salmonella | Serum | Tubex^®^ TF rapid typhoid detection, IDL Biotech AB, Bromma, Sweden; performed in laboratory of serology, Clinical Pathology Department, Hasan Sadikin General Hospital |
| PCR Leptospira or Salmonella | Whole blood | Laboratory of Medical Microbiology, Radboud University Medical Center, Nijmegen, Netherlands |
| C-reactive protein | Serum | CRPL3, C-Reactive Protein Gen3, Cobas (Roche Diagnostics GmbH, US); measurement range of 0.3-350 mg/L; performed in laboratory of chemistry, Clinical Pathology Department, Hasan Sadikin General Hospital |
| Procalcitonin | Serum | BRAHMS PCT, Elecsys and Cobas e 411 analyzers (Roche Diagnostics GmbH, US); measuring range of 0.02-100 ng/mL; performed in laboratory of serology, Clinical Pathology Department, Hasan Sadikin General Hospital |
