# Supplementary material for: A novel diagnostic algorithm equipped on an automated hematology analyzer to differentiate between common causes of febrile illness in Southeast Asia
Source: PLoS Negl Trop Dis. 2019 Mar 14;13(3):e0007183. doi: 10.1371/journal.pntd.0007183 (PMC6435198; doi:10.1371/journal.pntd.0007183)
Supplement: S2 Table — (DOCX) [file pntd.0007183.s002.docx]

**Supplemental information**

**S2 table. Baseline characteristics of proven-probable cases combined**

|  | **All patients**  **(n = 293)** | **Bacterial etiology**  **(n = 147)** | **Arboviral etiology**  **(n = 139)** | | | **Arboviral-bacterial**  **(n = 3)** | **Malaria**  **(n = 4)** |
| --- | --- | --- | --- | --- | --- | --- | --- |
| Age (year)* | 34.5 (20.2;50.8) | 47 (30.8;60.2) | 25 (19;38.5) | | | 17 (8.5;33) | 25 (19.8;35) |
| Male, n (%) | 129 (44) | 59 (40.1) | 67 (48.2) | | | 0 (0) | 3 (75) |
| Admitted, n (%) | 256 (87.4) | 124 (84.4) | 125 (89.9) | | | 3 (100) | 4 (100) |
| Fever, n (%) | 204 (69.6) | 84 (57.1) | 113 (81.3) | | | 3 (100) | 4 (100) |
| Duration of fever (days)* | 5 (3;7) | 5 (3;9.2) | 5 (3;5) | | | 8 (4.5;8.5) | 12 (8.5;12) |
| BMI (kg/m²)* | 20.8 (18.7;23.7) | 21 (18.5;24.2) | 20.8 (18.9;23.4) | | | 20 (18.4;22.5) | 23.2 (21.6;25) |
| Mortality, n (%) | 18 (6.1) | 17 (11.6) | 0 (0) | | | 1 (33.3) | 0 (0) |
| **Routine hematology** |  |  |  | | |  |  |
| White blood cells (WBC; 10³/µl)* | 6 (3.6;11.8) | 11.9 (7.3;17.4) | 3.8 (2.8;5.4) | | | 3.6 (3.5;4.3) | 4.7 (4.6;5.6) |
| Neutrophils (10³/µl)* | 3.3 (1.5;9) | 9.8 (5.4;15) | | 1.5 (1.1;2.4) | 2.6 (2.5;2.7) | | 2.2 (2.1;2.8) |
| Lymphocytes (10³/µl)* | 1.4 (0.8;2.1) | 1.3 (0.6;1.8) | | 1.7 (1;2.3) | 0.7 (0.6;1.2) | | 2.1 (1.7;2.6) |
| Monocytes (10³/µl)* | 0.5 (0.3;0.8) | 0.7 (0.4;1.1) | | 0.4 (0.2;0.6) | 0.4 (0.2;0.4) | | 0.5 (0.3;0.6) |
| Eosinophils (10³/µl)* | 0.02 (0.0;0.07) | 0.02 (0.0;0.08) | | 0.02 (0.0;0.05) | 0.02 (0.015;0.08) | | 0.07 (0.063;0.13) |
| Platelets (10^9^/l)* | 106 (54;230) | 207 (110;346) | | 72 (38;109) | 89 (59;96) | | 75 (56;95) |
| Hemoglobin (g/dl)* | 13.3 (11.2;15.1) | 11.4 (9.1;13) | | 14.8 (13.4;15.9) | 10.9 (10.4;13) | | 7.2 (6.4;8) |
| Hematocrit (%)* | 38.8 (33;43.9) | 33.5 (26.8;37.7) | | 42.8 (39.1;46) | 36.2 (32.3;40) | | 21.6 (18.2;25.3) |
| **Biomarkers** |  |  | |  |  | |  |
| CRP (mg/l)* | 28.7 (6.8;114.9) | 106.6 (51.3;190.2) | | 6.8 (3.6;17.2) | 96.6 (58.2;145.9) | | 142.3 (65;202.3) |
| PCT (ng/ml)* | 0.7 (0.3;2.6) | 1.8 (0.6;8.2) | | 0.4 (0.2;0.7) | 5 (3.8;8.8) | | 34.2 (20.7;43) |

Data are presented as median (25%; 75% percentile) unless indicated otherwise. CRP, C-reactive protein; PCR, procalcitonin
