# Supplementary figures and images for: A novel diagnostic algorithm equipped on an automated hematology analyzer to differentiate between common causes of febrile illness in Southeast Asia
Source: PLoS Negl Trop Dis. 2019 Mar 14;13(3):e0007183. doi: 10.1371/journal.pntd.0007183 (PMC6435198; doi:10.1371/journal.pntd.0007183)

**Supplemental Figures**

**S1 Fig. IMS algorithm**


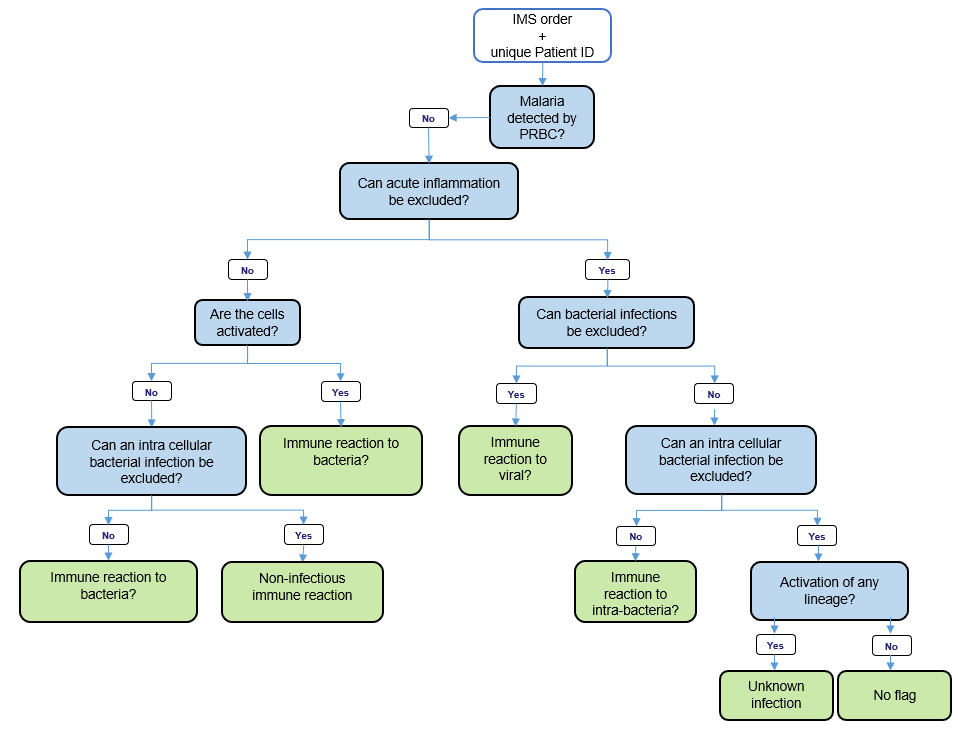

Supplement: S1 Fig — (DOCX) [file pntd.0007183.s003.docx]
