# Supplementary material for: A novel diagnostic algorithm equipped on an automated hematology analyzer to differentiate between common causes of febrile illness in Southeast Asia
Source: PLoS Negl Trop Dis. 2019 Mar 14;13(3):e0007183. doi: 10.1371/journal.pntd.0007183 (PMC6435198; doi:10.1371/journal.pntd.0007183)
Supplement: S1 Checklist — (DOC) [file pntd.0007183.s004.doc]

STROBE Statement—Checklist of items that should be included in reports of ***cohort studies***

|  | Item No | Recommendation |
| --- | --- | --- |
| **Title and abstract** | 1 | (*a*) Indicate the study’s design with a commonly used term in the title or the abstract. **Abstract, methodology first line** |
|  |  | (*b*) Provide in the abstract an informative and balanced summary of what was done and what was found, **Abstract** |
| Introduction | | |
| Background/rationale | 2 | Explain the scientific background and rationale for the investigation being reported. **Introduction, first two paragraphs.** |
| Objectives | 3 | State specific objectives, including any prespecified hypotheses. **Introduction, last paragraph** |
| Methods | | |
| Study design | 4 | Present key elements of study design early in the paper. **Methods, first paragraph, first line** |
| Setting | 5 | Describe the setting, locations, and relevant dates, including periods of recruitment, exposure, follow-up, and data collection. **Methods, first paragraph (design, study sites and populations)** |
| Participants | 6 | (*a*) Give the eligibility criteria, and the sources and methods of selection of participants. Describe methods of follow-up. **Methods, first and second paragraph (design, study sites and populations; study procedures)** |
|  |  | (*b*) For matched studies, give matching criteria and number of exposed and unexposed. **NA** |
| Variables | 7 | Clearly define all outcomes, exposures, predictors, potential confounders, and effect modifiers. Give diagnostic criteria, if applicable. **Methods, 3^rd^-5^th^ paragraph (Diagnostic procedures and case definitions; Laboratory procedures; Infection Manager System and analysis)** |
| Data sources/ measurement | 8* | For each variable of interest, give sources of data and details of methods of assessment (measurement). Describe comparability of assessment methods if there is more than one group. **Methods, 4^th^-5^th^ paragraph (Laboratory procedures; Infection Manager System and analysis)** |
| Bias | 9 | Describe any efforts to address potential sources of bias. **Methods, 2^nd^ paragraph** |
| Study size | 10 | Explain how the study size was arrived at. **Methods, first paragraph** |
| Quantitative variables | 11 | Explain how quantitative variables were handled in the analyses. If applicable, describe which groupings were chosen and why. **Methods, analysis paragraph** |
| Statistical methods | 12 | (*a*) Describe all statistical methods, including those used to control for confounding. **Methods, analysis paragraph** |
|  |  | (*b*) Describe any methods used to examine subgroups and interactions. **Methods, analysis paragraph** |
|  |  | (*c*) Explain how missing data were addressed. **Patients with missing data were excluded (results, paragraph 1)** |
|  |  | (*d*) If applicable, explain how loss to follow-up was addressed. **NA** |
|  |  | (*e*) Describe any sensitivity analyses. **Not performed** |
| Results | | |
| Participants | 13* | (a) Report numbers of individuals at each stage of study—eg numbers potentially eligible, examined for eligibility, confirmed eligible, included in the study, completing follow-up, and analysed. **Results paragraph 1,** **Figure 1 study flow** |
|  |  | (b) Give reasons for non-participation at each stage. **NA** |
|  |  | (c) Consider use of a flow diagram. **Figure 1** |
| Descriptive data | 14* | (a) Give characteristics of study participants (eg demographic, clinical, social) and information on exposures and potential confounders. **Results paragraph 1** |
|  |  | (b) Indicate number of participants with missing data for each variable of interest. **Patients with missing data were excluded (results, paragraph 1)** |
|  |  | (c) Summarise follow-up time (eg, average and total amount). **NA in this study** |
| Outcome data | 15* | Report numbers of outcome events or summary measures over time. **No data over time** |
| Main results | 16 | (*a*) Give unadjusted estimates and, if applicable, confounder-adjusted estimates and their precision (eg, 95% confidence interval). Make clear which confounders were adjusted for and why they were included. **Results 2^nd^ and 3th paragraph** |
|  |  | (*b*) Report category boundaries when continuous variables were categorized. **Results, 3th paragraph** |
|  |  | (*c*) If relevant, consider translating estimates of relative risk into absolute risk for a meaningful time period |
| Other analyses | 17 | Report other analyses done—eg analyses of subgroups and interactions, and sensitivity analyses. **No subgroup analyses done** |
| Discussion | | |
| Key results | 18 | Summarise key results with reference to study objectives. **Discussion, first paragraph** |
| Limitations | 19 | Discuss limitations of the study, taking into account sources of potential bias or imprecision. Discuss both direction and magnitude of any potential bias. **Discussion, 6^th^ paragraph** |
| Interpretation | 20 | Give a cautious overall interpretation of results considering objectives, limitations, multiplicity of analyses, results from similar studies, and other relevant evidence. **Discussion, last paragraph** |
| Generalisability | 21 | Discuss the generalisability (external validity) of the study results. **Discussion, section on limitations** |
| Other information | | |
| Funding | 22 | Give the source of funding and the role of the funders for the present study and, if applicable, for the original study on which the present article is based. **Information added in the submission system** |

*Give information separately for exposed and unexposed groups.

**Note:** An Explanation and Elaboration article discusses each checklist item and gives methodological background and published examples of transparent reporting. The STROBE checklist is best used in conjunction with this article (freely available on the Web sites of PLoS Medicine at http://www.plosmedicine.org/, Annals of Internal Medicine at http://www.annals.org/, and Epidemiology at http://www.epidem.com/). Information on the STROBE Initiative is available at http://www.strobe-statement.org.
